# Phagocytosis of Mature Granulocytes by Bone Marrow Macrophages in an Elderly Man with Adult-Onset Primary Autoimmune Neutropenia

**DOI:** 10.3390/hematolrep14020024

**Published:** 2022-05-25

**Authors:** Mitsutaka Nishimoto, Takahiko Nakane, Hideo Koh, Yasuhiro Nakashima, Ryosuke Yamamura, Hirohisa Nakamae, Masayuki Hino, Kensuke Ohta

**Affiliations:** 1 Hematology Ohta Clinic, Shinsaibashi, Osaka 5420081, Japan; nakane@med.osaka-cu.ac.jp (T.N.); yamamura@nakatsu.saiseikai.or.jp (R.Y.); ohtak@ligare-clinic.com (K.O.); 2 Hematology, Graduate School of Medicine, Osaka Metropolitan University, Osaka 5458585, Japan; hide_koh@med.osaka-cu.ac.jp (H.K.); yakkun414@med.osaka-cu.ac.jp (Y.N.); hirohisa@omu.ac.jp (H.N.); hinom@med.osaka-cu.ac.jp (M.H.)

**Keywords:** case report, autoimmune neutropenia, phagocytosis, bone marrow macrophage, prednisolone

## Abstract

Adult-onset primary autoimmune neutropenia (AIN) is an extremely rare but sometimes life-threatening disease. Its pathophysiology is still to be clarified. We describe a case with adult-onset primary AIN with phagocytosis of mature granulocytes by macrophages in bone marrow. A 77-year-old male was referred to our hospital with severe neutropenia. Based on the normal cellular bone marrow without morphological dysplasia and the positivity of anti-neutrophil antibodies in the serum, adult-onset primary AIN was diagnosed. After five years from the initiation of granulocyte colony-stimulating-factor therapy, neutropenia had progressed. At that time, the second bone marrow examination revealed segmented neutrophils phagocytosed by macrophages. Continuous low dose prednisolone succeeded to increase the neutrophil count. An impressive morphological feature of AIN indicated the destruction of mature granulocytes in bone marrow by antibody-dependent cellular phagocytosis mediated by granulocyte-specific antibodies. More cases should be accumulated to elucidate the precise mechanism and establish the optimal therapy.

## 1. Introduction

Primary autoimmune neutropenia (AIN) is a hematological disorder characterized by the destruction of neutrophils caused by granulocyte-specific antibodies [1,2,3]. It occurs predominantly in infancy and typically achieves spontaneous remission within a few months [4]. In contrast, adult-onset primary AIN is an extremely rare but sometimes life-threatening disease, suggesting differences in the pathophysiology between infants and adults.

It is well known that autoimmune antibodies, such as anti-human neutrophil antigen (anti-HNA) antibodies, are detected in the peripheral blood. However, the precise mechanism and optimal treatment for primary AIN are unclear.

We herein report a case of adult-onset primary AIN in which neutropenia was exacerbated by phagocytosis of mature granulocytes by macrophages in the bone marrow.

## 2. Case Report

A 77-year-old man with atrial fibrillation and hypertension was referred to our hospital due to neutropenia that had been gradually developing over the past 3 years. His laboratory data showed no significant abnormalities except for mild leukopenia and severe persistent neutropenia (Table 1). No manifestations indicative of autoimmune diseases, such as a skin rash, fever, arthritis, or splenomegaly, were observed on his physical examination. In addition, he had no familial history of neutropenia or myeloid malignancies. On clinical evaluation, there were no lymphoedema, warts, pulmonary disease, immunodeficiency, or monocytopenia. Bone marrow aspiration and a biopsy revealed normal cellularity without morphological dysplasia in hematological lineages. In the chromosomal analysis of bone marrow cells, 4 out of 20 analyzed cells showed the loss of chromosome Y, which seemed to be acquired with aging process [5]. Based on these findings, his neutropenia met the criteria of chronic primary neutropenia [6]. Anti-neutrophil antibodies in his serum were investigated with the six-cell lineage immunofluorescence test, monoclonal antibody-specific immobilization of granulocyte antigens [7], and microbeads assay using LABSCreen Multi (One Lambda, Inc., West Hills, CA, USA) [8] by the Japanese Red Cross Kinki Block Blood Center [9]. Specific IgM and IgG against HNA-1a and HNA-1d were detected and primary AIN was diagnosed.

Weekly subcutaneous injection of granulocyte colony-stimulating factor (G-CSF) had been initiated and succeeded in preventing severe infections. However, at five years after the diagnosis of primary AIN, the efficacy of the G-CSF therapy was dampened, and the neutrophil count remained low, causing repeated episodes of pneumonia. Although the symptoms such as fever, cough, and fatigue accompanied by pneumonia had been improved, neutrophil count remained low for more than two months after resolution of pneumonia.

**Figure 1:**
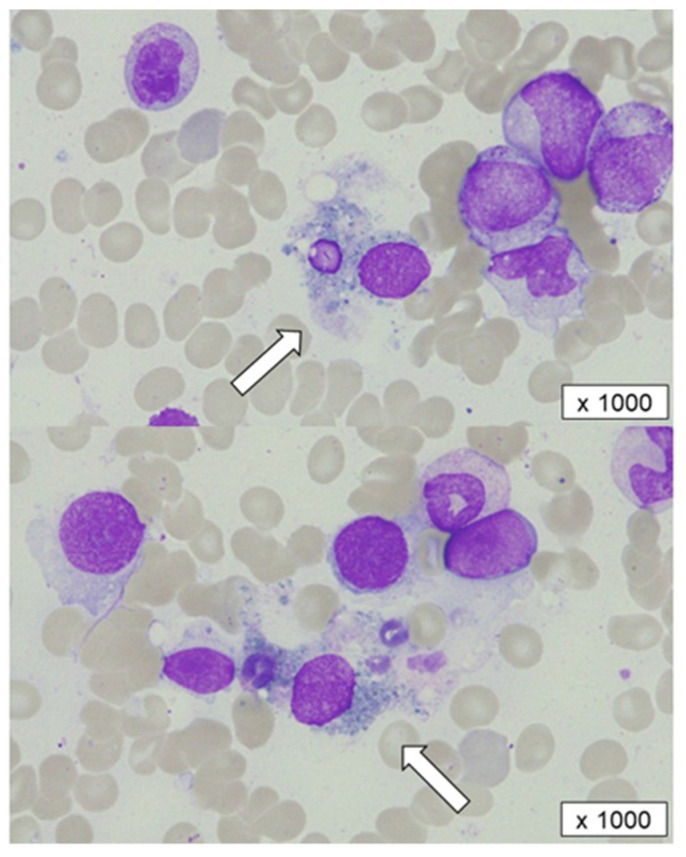
A bone marrow aspiration before corticosteroid therapy. Segmented cells phagocytosed and destroyed by macrophages (white arrows) were observed.

A second bone marrow examination showed slightly hypercellular marrow and a marked reduction in segmented neutrophils without morphological dysplasia. Notably, in his bone marrow, the segmented neutrophils—but not myeloid progenitor cells, platelets, or erythrocytes—had been phagocytosed and destroyed by macrophages, indicating the presence of specific antibodies for mature granulocytes (Figure 1). Anti-HNA antibodies in the serum remained positive at that time. Although the slight elevation of serum ferritin and soluble interleukin 2 receptor suggested the activation of immune cells, such as macrophages or lymphocytes, hemophagocytic syndrome was considered unlikely due to the lack of signs of platelets and hemoglobin decrement.

**Figure 2:**
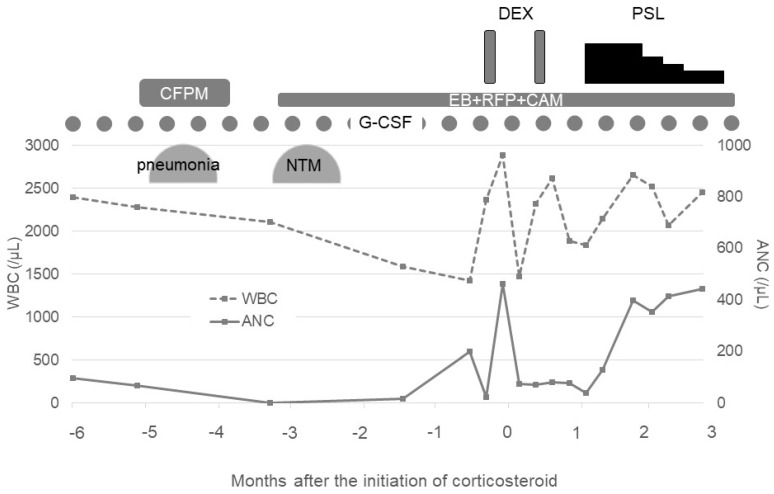
Clinical course before and after corticosteroid therapy. G-CSF: granulocyte colony-stimulating factor, CFPM: cefepime, NTM: nontuberculous mycobacteria, EB: ethambutol, RFP: rifampicin, CAM: clarithromycin, DEX: dexamethasone, PSL: prednisolone, WBC: white blood cell count, and ANC: absolute neutrophil count.

As the progression of AIN was suspected, oral prednisolone with 0.5 mg/kg was initiated after the failure of two courses of mini-pulse of dexamethasone with 20 mg for 4 days. The recovery of neutrophils was observed 14 days after the initiation of prednisolone therapy, and the dose of prednisolone started to be tapered. At present, his neutrophil count has been maintained for six months since the initiation of prednisolone therapy (Figure 2). Unfortunately, the changes in the anti-HNA antibody titers during the clinical course were unable to be examined.

## 3. Discussion

We encountered a rare case of adult-onset primary AIN in which neutropenia progressed with phagocytosis of granulocytes in the bone marrow and was successfully treated with low-dose prednisolone. To our knowledge, this is the first report of an adult case of AIN with impressive morphological features indicating the destruction of mature granulocytes in bone marrow by antibody-dependent cellular phagocytosis mediated by granulocyte-specific antibodies.

Mostly based on in vitro observations, the reduction in neutrophils in AIN has been speculated to be induced by three major mechanisms. The first possible mechanism is the simple agglutination of neutrophils mediated by anti-neutrophil antibodies. The second possible mechanism is complement-induced neutrophil agglutination, wherein complements activated by anti-neutrophil antibodies cause neutrophil aggregation and adherence to endothelial cells [3]. Rustagi et al. presented data on complement activation induced by anti-neutrophil antibodies in sera from 18 patients with systemic lupus erythematosus [10]. The third possible mechanism is phagocytosis of granulocytes. Although relatively rare, Bux et al. described the phagocytosis of granulocytes by bone marrow macrophages in 5 out of 240 cases of AIN in infancy [4]. Neutrophils opsonized by anti-neutrophil antibodies can also be ingested by splenic macrophages, as suggested by splenomegaly found in some cases of AIN [11]. Phagocytosis of the sensitized neutrophils can be considered to occur in tissues other than the marrow or spleen.

In the present case, phagocytosis of neutrophils appeared in bone marrow five years after the diagnosis of AIN, suggesting that the pathophysiology had changed over time. No other lineages of progenitor cells were phagocytosed, unlike the changes seen in hemophagocytic syndrome. Indeed, the mild elevations of serum ferritin and soluble interleukin 2 receptor supported the hypothesis that macrophages had been not “aberrantly” but “functionally” activated, and the neutrophils sensitized by specific anti-neutrophil antibodies had been digested by macrophages. In addition, antibiotics including anti-tuberculosis drugs for pneumonia might have had some influence on the exacerbation and persistence of neutropenia. However, the clinical course of neutropenia before the administration of antibiotics and the response to prednisolone suggest that the severe neutropenia was predominantly caused by an immunological mechanism with anti-neutrophil antibodies rather than a drug-induced response.

The efficacy of periodic subcutaneous injection of G-CSF for prophylaxis against infection was undetermined. As in vivo absorption of anti-neutrophil antibodies with newly produced neutrophils stimulated by G-CSF was considered to lead to the prevention of neutrophil destruction [1,2,3,12,13], periodic subcutaneous injection of G-CSF, in our case, might have contributed to the prevention of severe infections for five years. However, with the progression of phagocytosis of neutrophils in bone marrow as described above, the persistence of severe neutropenia caused repeated episodes of pneumonia. The mechanism underlying this progression in our case was unclear. However, while we were unable to rigorously examine anti-HNA antibodies again in the progressive phase, increased titers and/or the development of different types of antibodies seemed to be responsible for the progression.

Interestingly, this exacerbation of neutropenia was successfully improved by continuous low-dose prednisolone therapy over fourteen days, in accordance with the previous reports [14,15,16,17], but not by short-term dexamethasone therapy. Immunosuppressant agents have been used for G-CSF refractory AIN [14,15,16,17,18,19,20,21,22,23]. Successful treatment with a single use of corticosteroid or combination with cyclosporine has been reported in several cases of AIN with [14,15,18] or without [16,17,18,19,20] other lineages of cytopenia, such as autoimmune hemolytic anemia or idiopathic thrombocytopenic purpura. The response has been observed around two to three weeks after the initiation of corticosteroid therapy, and the efficacy has lasted even after discontinuation of the therapy. 

We initially treated with short-term dexamethasone instead of continuous low-dose prednisolone therapy to prevent the adverse effects caused by long-term use of corticosteroid. However, the poor response to short-term high-dose dexamethasone in our case suggested that the pathophysiology of AIN should be different from that of idiopathic thrombocytopenic purpura, in which promising outcomes have been reported with a rapid response resulting in less-adverse effects, including infections, than with continuous prednisolone [21]. Similarly, unlike ITP, AIN does not respond to treatment with rituximab that selectively targets B lymphocytes [22]. Taken together, these findings indicate that for the treatment of AIN patients, it may be necessary to control not only antibodies but also extensive immune cells that can aid in the expansion of antibody-producing autoreactive B lymphocytes. This hypothesis is supported by the durable response of AIN to treatment with alemtuzumab, an anti-CD52 antibody that targets T lymphocytes, macrophages, monocytes, and dendritic cells [23,24]. Profound and broad immunosuppression by alemtuzumab, increasing the risk of infection, can be more effective for cases with refractory AIN.

## 4. Conclusions

We encountered a rare case of adult-onset primary AIN successfully treated with prednisolone. Phagocytosis by macrophages in the bone marrow was likely related to his neutropenia. To clarify the precise mechanism and establish optimal therapy for adult-onset primary AIN, the further accumulation of cases is warranted.

## Figures and Tables

**Table 1:** Laboratory and bone marrow findings at the diagnosis and before corticosteroid therapy.

		At Diagnosis	Before Corticosteroid
**Peripheral blood**			
WBC	(/µL)	1900	1590
Stab cell	(%)	4.0	1.0
Segmented cell	(%)	4.0	0.0
Eosinophil	(%)	11.0	30.0
Basophil	(%)	1.0	3.0
Monocyte	(%)	20.0	26.0
Lymphocyte	(%)	60.0	40.0
RBC	(/µL)	458 × 10^4^	403 × 10^4^
Hemoglobin	(g/dL)	13.4	12.5
Platelet	(/µL)	22.5 × 10^4^	12.8 × 10^4^
Reticulocyte	(‰)	18.3	
Total bilirubin	(mg/dL)	0.5	0.4
AST	(IU/L)	23	20
ALT	(IU/L)	20	17
LDH	(IU/L)	160	157
γ-GTP	(IU/L)	23	31
Creatinine	(mg/dL)	1.0	0.92
Vitamin B12	(pg/mL)	462	
Folic acid	(ng/mL)	5.8	
IgG	(mg/dL)		1930
IgA	(mg/dL)		329
IgM	(mg/dL)		77
CH50	(U/mL)		50.9
ANA		×40	×40
Anti-ds DNA	(IU/mL)		<2.0
sIL-2R	(U/mL)		1181
Ferritin	(ng/mL)		221
C-reactive protein	(mg/dL)	1.39	1.60
**Bone marrow**			
Total nucleated cells	(/µL)	102,000	176,000
Myeloblast	(%)	0.0	2.0
Promyelocyte	(%)	0.0	4.6
Myelocyte	(%)	16.0	18.2
Metamyelocyte	(%)	13.0	13.6
Stab cell	(%)	23.4	23.8
Segmented cell	(%)	5.2	2.8
Eosinophil	(%)	2.8	5.4
Basophil	(%)	0.2	0.4
Monocyte	(%)	2.8	3.2
Lymphocyte	(%)	11.2	8.0
Plasma cell	(%)	1.4	2.0
Proerythroblast	(%)	0.0	0.2
Baso erythroblast	(%)	0.4	1.2
Poly erythroblast	(%)	23.2	12.8
Ortho erythroblast	(%)	0.0	0.6
G-Band		45,X,-Y [4/20] 46,XY [16/20]	45,X,-Y [3/20] 46,XY [17/20]

ALT: alanine transferase, ANA: anti-nuclear antibody, AST: aspartate aminotransferase, Baso: basophilic, CH50: 50% hemolytic complemental activity, γ-GTP: gamma glutamyl transferase, LDH: lactate dehydrogenase, Ortho: orthochromatic, Poly: polychromatic, RBC: red blood cells, sIL-2R: soluble interleukin 2 receptor, and WBC: white blood cells.

## Data Availability

The data that support the findings of this case report are available from the corresponding author, M.N., upon reasonable request.
